# When the tap runs dry: the physiological effects of acute experimental dehydration in *Peromyscus eremicus*

**DOI:** 10.1242/jeb.246386

**Published:** 2023-12-01

**Authors:** Danielle M. Blumstein, Matthew D. MacManes

**Affiliations:** Department of Molecular, Cellular, and Biomedical Sciences, University of New Hampshire, Durham, NH 03824, USA

**Keywords:** Energy expenditure, *Peromyscus*, Physiology, Respirometry, Total water loss, Dehydration

## Abstract

Desert organisms have evolved physiological, biochemical and genomic mechanisms to survive the extreme aridity of desert environments. Studying desert-adapted species provides a unique opportunity to investigate the survival strategies employed by organisms in some of the harshest habitats on Earth. Two of the primary challenges faced in desert environments are maintaining water balance and thermoregulation. We collected data in a simulated desert environment and a captive colony of cactus mice (*Peromyscus eremicus*) and used lab-based experiments with real time physiological measurements; energy expenditure, water loss rate and respiratory exchange rate, to characterize the response to water deprivation. Mice without access to water had significantly lower energy expenditures and in turn, reduced water loss compared to mice with access to water after the first 24 h of the experiment. Additionally, we observed significant mass loss that is probably due to dehydration-associated anorexia a response to limit fluid loss by reducing waste and the solute load as well as allowing water reabsorption from the kidneys and gastrointestinal tract. Finally, we observed body temperature correlated with sex, with males without access to water maintaining body temperature when compared with hydrated males, whereas body temperature decreased for females without access to water, suggesting daily metabolic depression in females.

## Teaser

**Highlighted Article:** Cactus mice exhibit dehydration anorexia, which is an adaptive response aimed at limiting fluid loss by reducing waste and solute load, enabling them to survive in desert habitats.

## INTRODUCTION

Water is arguably the single most important factor for life on Earth, and in organisms, water is stored in intracellular and extracellular spaces (Fitzsimons, 1963). Dehydration occurs where there is a decrease in extracellular fluid volume caused when the loss is outpaced by fluid intake and metabolic water production, leading to a negative fluid balance and increased serum osmolality (Thomas et al., 2008). Regardless of the habitat, animals must regulate body fluids to protect against or cope with dehydration (Takei, 2015). Mammals have developed many different mechanisms for body fluid regulation (Christian and Matson, 1978; Frank, 1988; Jirimutu et al., 2012; Marra et al., 2014; Yang et al., 2016; Kordonowy et al., 2017) and these mechanisms could aid in survival given that the most well-supported climate change scenarios predict increased aridity (Mirzabaev and Wu, 2019).

Climate change is rapidly reshaping habitats globally and is predicted to continue (Hughes, 2000; Parmesan and Yohe, 2003; Parmesan, 2006), modifying selective pressures for many populations (Hochachka and Somero, 2002; Pörtner, 2002; Pörtner and Farrell, 2008). Understanding environmental tolerance and the capacity for adaptation in one species can provide insight into the potential for similar species to respond to increasingly extreme climatic patterns. In recent years, many habitats have recorded some of the hottest temperatures to date (IPCC, 2019; Stillman, 2019), resulting in environmental thresholds that may exceed organismal tolerance. Furthermore, climate change is increasing global desertification rates, increasing water stress among wildlife (Loarie et al., 2009; Mirzabaev and Wu, 2019). To maintain viable populations, organisms must survive and successfully reproduce under climate warming and aridification by using existing phenotypes and phenotypic plasticity, rapid evolution or geographic range and phenological shifts (Hofmann and Todgham, 2010; Hoffmann and Sgrò, 2011; Brown et al., 2016). Despite climate change altering habitats and impacting populations, habitat distributions for rodents have remained remarkably stable over the last century of climate change, largely because of behavioral changes (Moritz et al., 2008; Pardi et al., 2020; Riddell et al., 2021).

Desert habitats are characterized by an extreme lack of precipitation, which exerts a controlling effect on biological processes (Rocha et al., 2021). Daily temperatures in the Sonoran Desert can fluctuate by as much as 30–50°C (Reid, 1987; Sheppard et al., 2002). During the summer months, temperatures can reach upwards of 50°C during the day, while at night they may drop to as low as 15°C (Coppernoll-Houston and Potter, 2018). In the winter, daytime temperatures are typically between 20 and 30°C, whereas night-time temperatures can drop to near freezing (Boyd Deep Canyon Desert Research Center). Daily rainfall in the Sonoran Desert is relatively rare, with most areas receiving less than a centimeter of rain per year (Boyd Deep Canyon Desert Research Center)**.** Organisms that are adapted to live in desert habitats must manage their water budgets over long dry and hot periods of time.

Organisms maintain water homeostasis in several ways, including seeking out sources of free-flowing water, preformed dietary water (Frank, 1988; Orr et al., 2015) and/or producing water via metabolism. However, if adequate water is not acquired, dehydration can negatively affect an animal's ability to regulate its body temperature, impair its cardiovascular function and decrease perfusion to organ systems. Specifically, dehydration results in a decrease in blood volume and increase in osmolality, primarily driven by the increase in serum sodium levels (Leib et al., 2016; Thornton, 2010). As a result, several neurohormonal systems are activated to maintain blood pressure to perfuse tissues appropriately (Kaufmann et al., 2020). Water is recovered in the gastrointestinal tract (Thiagarajah and Verkman, 2018) and in the kidneys it is reabsorbed from the tubule system back into the blood stream (Fuller et al., 2020; Kortenoeven and Fenton, 2014). In severe cases, dehydration can lead to organ failure and death.

Mice of the genus *Peromyscus* have the widest distribution of any North American mammal and have unparalleled habitat diversity (Bedford and Hoekstra, 2015). Several members of the genus, including the cactus mouse (*Peromyscus eremicus*) are native inhabitants of the arid deserts in southwest North America, including the Sonoran Desert (Murie, 1961; Pavlik, 2008). Past studies have shown that *Peromyscus eremicus* are extremely efficient at water retention by limiting water loss during excretion and maximizing water reabsorption (Schmidt-Nielsen and Adolph, 1964; Schmidt-Nielsen and Schmidt-Nielsen, 1952) with little to no renal impairment (Kordonowy et al., 2017) and they show limited tissue damage when dehydrated (MacManes, 2017). *Peromyscus eremicus* also have the slowest metabolism amongst the *Peromyscus* species (Mueller and Diamond, 2001) and very high heat tolerance, with thermal limits between 43.4°C and 45.6°C (Ramirez et al., 2022). Furthermore, these mice have a suite of genomic adaptations such as genes involved in maintaining water and salt balance (Colella et al., 2021a; Kordonowy and MacManes, 2016; MacManes, 2017; MacManes and Eisen, 2014) and selection for genes consistent with adaptations to life in the desert (Tigano et al., 2020; Colella et al., 2021a). However, unlike *Dipodomys* sp., *P. eremicus* do not have kidney modifications (i.e. elongated loops of Henle) (Dewey et al., 1966; MacManes, 2017) and are not skilled burrowers, primarily nesting in rock heaps and cavities, resulting in a minimal microclimate effect (Hu and Hoekstra, 2017; Weber and Hoekstra, 2009). Animals of this genus can be held in captivity (Crossland et al., 2014), have extensive genomic resources (Colella et al., 2021a; Tigano et al., 2020) and there are a wealth of samples collected historically and contemporarily in natural history museums (Pergams and Lawler, 2009). Together, these features make *P. eremicus* ideal for investigating water economy.

Here, we expand on the long history of studies of organismal water management in desert taxa (Albright et al., 2017; Blumstein et al., 2022 preprint; Bradford, 1974; Cortés et al., 2000; Frank, 1988; Hayes et al., 1998; Kordonowy et al., 2017; MacMillen, 1983; Schmidt-Nielsen and Schmidt-Nielsen, 1952, 1952) to assess the physiological response to water deprivation in a hot and dry environment. To accomplish this, we compared multiple physiological responses, rate of water loss, energy expenditure, respiratory quotient, a suite of electrolytes, body mass and body temperature, for mice with and without access to water for 72 h to understand how animals survive the extreme head and aridity of deserts and further characterize the response of *P. eremicus* to water deprivation.

## MATERIALS AND METHODS

### Animal care and experimental model

All animals used in this study were captive born, sexually mature, non-reproductive healthy adult male and female *Peromyscus eremicus* (Baird 1858). Individuals were descended from wild caught animals from a hot/dry-desert population in Arizona collected in 1993 and maintained at the Peromyscus Genetic Stock Center (Columbia, SC, USA). The University of New Hampshire colony was founded in 2013 with 40 mice and supplemented by 30 more individuals in 2018. All *P. eremicus* in University of New Hampshire colony are bred and housed in a large walk-in environmental chamber designed to simulate the temperature, humidity and photoperiod of their native desert environment (Kordonowy et al., 2017; Colella et al., 2021b; Blumstein et al., 2022 preprint). The daytime (light) phase lasted for 12 h (08:00 h to 20:00 h) at a room temperature of 32°C and 10% RH followed by a 1 h transition period where the temperature consistently decreased and the RH increased until reaching a room temperature of 24°C and 25% RH for the night-time (dark) phase which lasted for 9 h (21:00 h to 06:00 h). To compete the cycle, there was a 2 h transition period where the temperature consistently increased and RH decreased until returning the room to light phase conditions (Kordonowy et al., 2017; Colella et al., 2021b; Blumstein et al., 2022 preprint). Animal care procedures were approved by the University of New Hampshire Institutional Animal Care and Use Committee under protocol number 210,602 and followed guidelines established by the American Society of Mammologists (Sikes and Gannon, 2011). Mice were provided a standard diet and fed *ad libitum* [LabDiet^®^ 5015*; 26.101% fat, 19.752% protein, 54.148% carbohydrates, energy 15.02 kJ g^−1^, food quotient (FQ) 0.89].

Prior to experimental treatment mice were weighed (rounded to the nearest tenth of a gram) on a digital scale. A temperature-sensing passive integrated transponder (PIT) tag (BioThermo13, accuracy ±0.5°C, BioMark^®^, Boise, ID, USA) was implanted subdermally between the shoulders, per the University of New Hampshire veterinary staff recommendations, using a tag injector (Biomark^®^ MK10). Animals were then allowed to recover individually in an experimental chamber for 1 week of observation before the experiments were started. Body temperature was recorded at noon and midnight via a Biomark^®^ HPR Plus reader and mass was measured at noon every day over the duration of the experiment. A randomly selected set of animals was assigned to the two water treatment groups (*n*=9 of each treatment, female mice with water, female mice without water, male mice with water, and male mice without water, total *n*=36). At the start of the experiment (day 0, time 0, 10:00 h), water was removed from three of the chambers corresponding to those animals in the dehydration group. No mortality occurred during these experiments. Three days later, at the conclusion of the experiment, mice were euthanized via isoflurane overdose and decapitation, and we collected 120 µl of trunk blood for serum electrolyte measurement using an Abaxis i-STAT^®^ Alinity machine. Using i-STAT CHEM8+ cartridges (Abbott Park, IL, USA, Abbott Point of Care Inc), we measured the concentration of sodium (Na, mmol l^−1^), potassium (K, mmol l^−1^), blood urea nitrogen (BUN, mmol l^−1^), hematocrit (Hct, % PCV), ionized calcium (iCa, mmol l^−1^), glucose (Glu, mmol l^−1^), osmolality (mmol l^−1^), hemoglobin (Hb, g dl^−1^), chlorine (Cl, mEq l^−1^), total CO_2_ (T_CO_2__, mmol l^−1^) and Anion gap (AnGap, mEq l^−1^). Using Na, Glu, and BUN, we calculated serum osmolality. The experimental setup was repeated six times for three male batches and three female batches.

### Metabolic phenotyping

During the experiment mice were exposed to either water deprivation or control conditions where water was provided *ad libitum* for three continuous days while being housed in transparent 9.5 l respirometry chambers with dried cellulose-based bedding. Air was continuously pulled from the chambers using a pull flow-through respirometry system from Sable Systems International (SSI) starting with SS-4 Sub-Sampler Pumps, one for each chamber, at a rate of 1600 ml min^−1^ (96 l h^−1^). The SSI MUXSCAN was used to multiplexed air streams, alternating between a baseline (empty) chamber measurement for 120 s and a random animal chamber measurement for 120 s each, measuring each chamber 120 s approximately twice every hour. Finally, the Field Metabolic System (FMS, zeroed and spanned between each 72 h experiment using dry gas with known concentrations of CO_2_ and O_2_) sub-sampled the airstream at 250 ml min^−1^ and measured temperature (°C), barometric pressure (kPa), water vapor (kPa), CO_2_ and O_2_, with no scrubbing.

### Calculations and statistical analysis

We analyzed our data using methods fully described in Colella et al. (2021b) and Blumstein et al. (2022). Rates of CO_2_ production, O_2_ consumption and water loss were calculated using Eqns 10.6, 10.5 and 10.9, respectively, from Lighton (2018). Respiratory exchange ratio (RER, the ratio of *V_CO2_* to *V_O2_*) and energy expenditure (EE) kJ h^−1^ was calculated as in Lighton (2018, Eqn 9.15). All downstream statistical analyses were conducted in R v.4.0.3 (https://www.r-project.org/). The R package mgcv::gamm (https://CRAN.R-project.org/package=mgcv) was used and included the fixed effects; access to water and sex, and interacting nonlinear smoothing regression terms with pairwise fixed effect combinations as interactions; time in days and diurnal cycle (Lin and Zhang, 1999; Wood, 2017) and visualized using gratia (Simpson, 2023). Experimental batches and the mice nested within the experimental batch were used as random effects to ensure we were not violating the assumption of independence. This allowed us to explain the average differences between groups of mice instead of explaining differences between individual mice. To test for statistical significant (*P*<0.05) differences in electrolytes after the treatments were applied and for each time point mass and body temperature were collected we used a Student's two-tailed *t*-test (stats::t.test) between the sexes for each experimental group.

## RESULTS

### Water loss rate

**Fig. 1.**
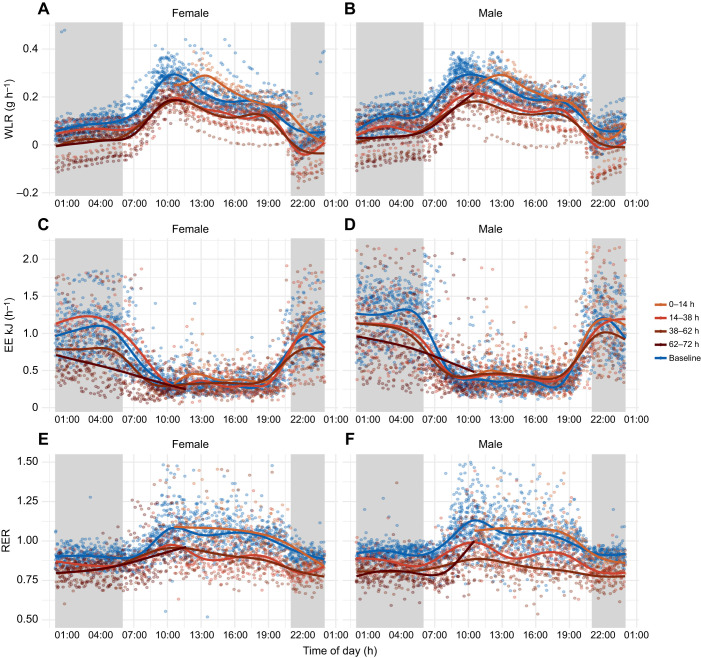
**Diurnal patterning of water loss rate, energy expenditure and respiratory exchange ratio in *Peromyscus eremicus*.** Seventy-two hours of respirometry data collection for 18 adult females (A,C,E) and 18 adult males (B,D,F) (*n*=9 of each treatment, total *n*=36) plotted in a 24 h window to display circadian patterns for each group. Baseline measurements of mice with water access (blue) and measurements of mice without access to water for 0–3 days (four shades of brown). Shaded sections in gray indicate the dark phase when animals are active, and unshaded blocks indicate light phase when animals are inactive. (A,B) 24 h water loss rate (WLR, H_2_O g h^−1^), (C,D) energy expenditure (EE, kJ h^−1^) and (E,F) respiratory exchange ratio (RER) for females and males.

Both experimental groups, water access and water deprivation, had diurnal patterning of water loss rate (WLR) with the highest occurring during the light phase and lowest during the dark phase (Fig. 1A,B). Each day of the experiment had similar patterns regardless of the treatment. During the first day, WLR was higher in males without access to water. For days two and three, both males and females without access to water had lower WLR (Fig. 1A,B).

Generalized additive modeling (GAM) analysis explained 77.2% of the deviance in WLR (Fig. S1, Table S1). Significant predictors of WLR included sex (*P*<0.0001) and water access (*P*<0.0001) but not sex×water access (*P*=0.13). All treatment combination splines were significant (Table S1). For both males and females without access to water, the curves for time in days by 24 h cycle were very complex, oscillating with the light:dark cycle and decreasing over time (Fig. 1A,B). The curves for time in days for males and females with access to water also oscillated with the light:dark cycle (Fig. 1A,B). Generally, mice without water had higher WLR during the first 24 h and lower WLR for the remainder of the experiment, based on GAM analysis and visualization (Fig. S1, Table S1, Fig. 1A,B). When comparing the four curves (males without water, males with water, females without water, females with water), WLR in mice without water was similar to that in mice with access to water: it converged during the light to dark transition phases, with the exception of the first transition (Fig. S1).

### Energy expenditure

Males and females in both experimental groups showed diurnal pattering, with the highest EE occurring during the dark (active) phase and the lowest EE occurring during the light (inactive) phase (Fig. 1C,D). This pattern continued over each day of the experiment for males and females with and without water.

During the first 24 h, EE was highest for females compared with males without water and with all mice with access to water. EE decreased over the subsequent 48 h for mice without access to water; females with without access to water had the lowest EE compared with males without access to water and all mice with access to water during the dark phase of days two and three of the experiment (Fig. S2, Table S2, Fig. 1C,D). Mice were manually weighed at 12:00 h every day, resulting in a transient increase of EE at that time (Fig. 1C,D). The GAM analysis explained 61.4% of the deviance in EE with significant predictors being sex (*P*<0.0001), water access (*P*<0.0001) and sex×water access (*P*=0.0464). All treatment combination splines were significant (Table S2).

### Respiratory exchange ratio

RER also showed diurnal patterning for both experimental groups and for both sexes (Fig. 1E,F). RER was highest during the light phases (Fig. 1E,F) and lowest and comparable to the FQ during the dark phases (Fig. 1E,F). Of the deviance, 43.3% was explained in the GAM analysis, with significant predictors being sex (*P*<2^−16^), water access (*P*=4.14^−08^) and the interaction between sex and access to water (*P*=1.41^−07^). All treatment combination splines were significant (Table S3) and complex, oscillating with the light:dark cycle (Fig. 1E,F, Fig. S3). Males and females without water access had higher RER compared with mice with water access during the first 24 h, based on GAM analysis and visualization (Fig. S3, Table S3). Interestingly, males without access to water had the lowest RER of any group over the course of the entire experiment during the second dark phase and for the remainder of the experiment (Fig. S3).

### Electrolytes, mass and body temperature

**Fig. 2.**
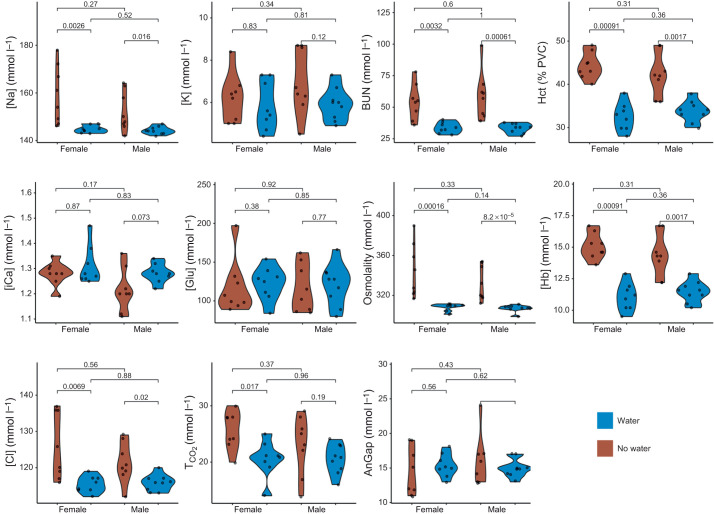
**Serum electrolytes in *P. eremicus* with or without access to water.** Violin plots showing the distribution of serum electrolytes, for female and male mice with (blue) or without (brown) access to water for 72 h**.** Observations (*n*=9 for each treatment; total *n*=36) are represented by black dots. *P*-values from pairwise *t*-tests are reported above the brackets. Na, sodium (mmol l^−1^); K, potassium (mmol l^−1^); BUN, blood urea nitrogen (mmol l^−1^); Hct, hematocrit (% PCV); iCa, ionized calcium (mmol l^−1^); Glu, glucose (mmol l^−1^); osmolality (mmol l^−1^); Hb, hemoglobin (g dl^−1^); Cl, chlorine (mmol l^−1^); T_CO_2__, total CO_2_ (mmol l^−1^); AnGap, anion gap (mmol l^−1^).

Several electrolytes were significantly different when comparing males with and without access to water and females with and without access to water (male and female: Na, *P*=0.0016 and *P*=0.0026 respectively; BUN, *P*=0.001 and *P*=0.003; Hct, *P*=0.002 and *P*=0.001; osmolality, *P*=8.2^−05^ and *P*=0.0001; Cl, *P*=0.02 and *P*=0.007; Hb, *P*=0.017 and *P*=0.009; T_CO_2__ female, *P*=0.017) (Fig. 2). No electrolytes were significantly different when comparing males with females for either water treatment (Fig. 2).

**Fig. 3.**
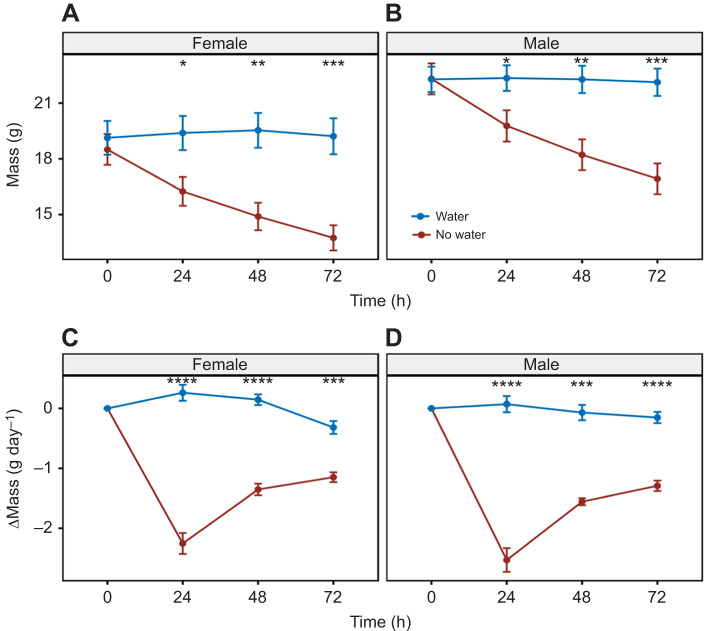
**Body mass changes in *P. eremicus* with or without access to water.** Body mass (A,B) and change in body mass (C,D) for female and male *P. eremicus* with (blue) or without (brown) access to water every 24 h over the course of the 72 h experiment. Data are means±s.e.m. Asterisks denote statistical significance from *t*-tests between the two treatments, with and without water (*n*=9 of each treatment, total *n*=36) at each time point (*P≤0.05, **P≤0.01, ***P≤0.001, ****P≤0.0001).

Although body mass of males and females did not differ significantly at the beginning of the experiment, males had a higher mass and both sexes lost mass over the course of the water deprivation experiment with the most mass loss occurring in the first 24 h without water (Fig. 3). When comparing mice with and without access to water within sexes, mice without water weighed significantly less than mice with access to water at 24 h (male and female: *P*=0.024 and *P*=0.019), 48 h (*P*=0.004 and *P*=0.002) and 72 h (*P*=0.001 and *P*=0.005) (Fig. 3A,B). Only animals held without water lost mass (Fig. 3C,D) and analysis of these changes showed that they were significantly different at all timepoints after water had been removed (24 h, *P*=4.1^−05^ and *P*=4.1^−05^; 48 h, *P*=0.001 and *P*=4.1^−05^; 72 h, *P*=4.1^−05^ and *P*=0.001).

**Fig. 4.**
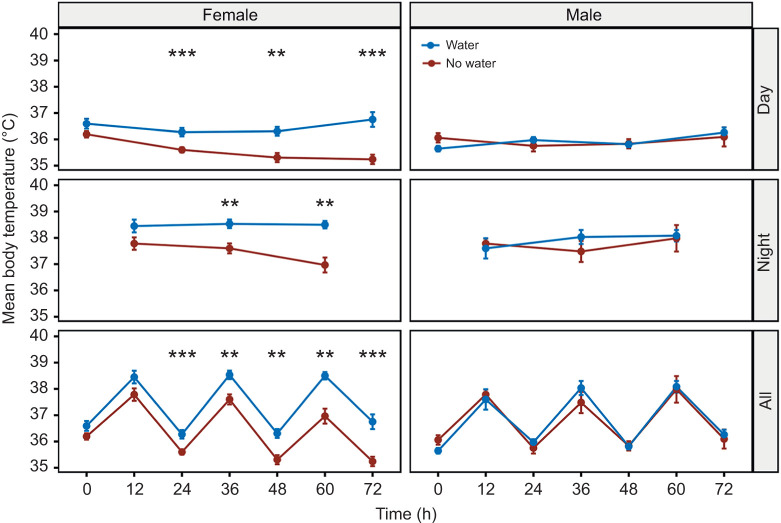
**Body temperature changes in *P. eremicus* with or without access to water.** Body temperatures for female and male *P. eremicus* with (blue) or without (brown) access to water (*n*=9 of each treatment, total *n*=36) every 12 h over the course of the 72 h experiment. The top row of graphs are measurements taken only during the light phase, middle row are measurements taken only during the dark phase, and bottom row represents all the measurements. Data are means±s.e.m. Asterisks denote statistical significance from *t*-tests between the two treatments, with and without water, at each time point (*P≤0.05, **P≤0.01, ***P≤0.001, ****P≤0.0001).

Body temperature showed diurnal pattering with the highest body temperature during the dark (active) phase and the lowest during the light phase (Fig. 4). For females, body temperature followed a similar pattern as described above and was significantly lower for mice without access to water at 24 h (*P*=0.001), 36 h (*P*=0.005), 48 h (*P*=0.001), 60 h (*P*=0.002) and 72 h (*P*=0.0003) while males were not significantly different at any of the time points (Fig. 4).

## DISCUSSION

We explored the relationships and trade-offs between thermoregulation, osmoregulation and energy expenditure of desert-adapted mice without access to drinking water for three days while housed in an environmental chamber that simulated the desert environment. We observed that when water was removed, energy expenditure and evaporative water loss are reduced in both sexes (presumably to conserve body water) at the expense of homeothermy, resulting in lower core body temperature in females but not in males. Though it may save water and/or energy, these physiological shifts could ultimately increase the risk of mortality and decrease fitness if water continues to be unavailable for extended periods of time.

### Mass loss and water deprivation

Our study targeted responses to water deprivation, investigating how physiological variables changed in response to dehydration throughout the course of the experiment. We found that in response to water deprivation, *P. eremicus* phenotypic responses changed rapidly. During the first 24 h of the water-deprivation experiment both males and females increased EE, resulting in an increase in WLR and a significant decrease in body mass. The reasons behind this dramatic shift are unclear but may be a result of (1) a behavioral response related to searching for drinking water and/or (2) suppression of food intake as suggested by anecdotal observations.

The relationship between eating and drinking has been extensively studied (Fitzsimons and Le Magnen, 1969; Kissileff, 1969; Smith, 2000; Watts, 1998; Zorrilla et al., 2005) and it has been documented that dehydration anorexia is an adaptive response to limit fluid loss (Watts and Boyle, 2010) as typically, the processing of food requires the use of water. Previous studies have demonstrated that dehydrated animals with *ad libitum* food match the same attributes of food-restricted animals, such as expression of hypothalamic neuropeptide genes, leptin and insulin levels, and corticosterone concentrations (Watts et al., 1999). Furthermore, the reduction of food intake results in a series of adaptive responses that target GI function, allowing for the absorption of the osmotically sequestered water that is normally in the GI (Kutscher, 1968; Lepkovsky et al., 1957; Schoorlemmer and Evered, 1993). Finally, reduced food intake also reduces the solute load (Rowland, 2007) and the need for removal of waste products via urinary water loss (Schoorlemmer and Evered, 1993). In our study, several tissues, including the GI tract, were removed at the conclusion of the experiment for future RNAseq analysis, and the GI tract was empty of food and feces (our unpublished observations), suggesting that the intake of solid food had decreased dramatically. Future studies may aim to quantify this observation as we saw a significant decrease in mass during the first 24 h of the experiment; however, the WLR during the first 24 h is not enough to account for the mass loss, suggesting mass loss through other means such as dehydration anorexia (Armstrong et al., 1980; Hamilton and Flaherty, 1973).

Previous studies have found that access to water (Hochman and Kotler, 2006; Shrader et al., 2008; Levy et al., 2016) and specific dietary composition (Blumstein et al., 2022 preprint; Frank, 1988; Manlick et al., 2021; Orr et al., 2015; Schmidt-Nielsen, 1975; Schmidt-Nielsen and Adolph, 1964; Wolf and del Rio, 2003) strongly affects populations living in arid environments. These external factors influence species distributions (McKee et al., 2015), modifying foraging decisions (Gedir et al., 2016, 2020) and altering behavior and reproduction (Douglas, 2001; McKinney et al., 2001; Cain et al., 2008). In the wild, cactus mice have been documented shifting diet seasonally, consuming arthropods during the winter (Hope and Parmenter, 2007) and transitioning to the consumption of cactus seeds and/or fruits during the summer (Hope and Parmenter, 2007; Orr et al., 2015). In addition to preformed water, the composition of the diet is also very important for *P. eremicus* as described by Blumstein et al. (2022). Specifically, mice fed a diet low in fat with *ad libitum* water lost significantly more water and had electrolyte levels suggesting dehydration compared with mice fed a diet higher in fat, suggesting a limited capacity to tolerate water deprivation if optimal foods become less abundant (Blumstein et al., 2022 preprint). Furthermore, the temperatures required to balance evaporative water loss with metabolic water production on dry seed are much lower than what occurs during the summers in desert regions (MacMillen and Hinds, 1983; Walsberg, 2000), suggesting that *P. eremicus* may not be able to survive on a only a dry diet, unlike the Heteromyids, which survive on dry diets alone (Frank, 1988; Schmidt-Nielsen et al., 1948).

Consistent with predictions of altered physiology and behavior mediated by water restriction, we recorded a decrease in EE, WLR, body mass and body temperature, and a shift in serum electrolytes in water-deprived *P. eremicus* during all three 24 h time blocks. While males and females without access to water had different magnitudes of change in EE and WLR throughout the duration of the study, both metabolic rates and the rate at which water is lost decreased, similarly to that recorded in other desert organisms (Schmidt-Nielsen et al., 1967; Taylor, 1969; Finch and King, 1982). EE and WLR are inherently related in animals as lower EE typically leads to lower water loss by decreasing the amount of dry air passing through the respiratory track (McFarlane and Howard, 1972).

### Water homeostasis and diet

Furthermore, catabolism of different diets varies in the amount of available energy (Sánchez-Peña et al., 2017) and water potential, as well as obligatory water loss (Schmidt-Nielsen, 1975). Specifically, the oxidation of carbohydrates yields a net metabolic water gain, whereas the oxidation of proteins results in net water loss, mainly through urination which is required to remove products of their metabolism, such as urea (Frank, 1988). In contrast, the impact of lipid oxidation on water is dependent on RH (Frank, 1988). At lower humidity (∼20% RH), lipid catabolism results in a slight net metabolic water loss, whereas at higher humidity (for example within a nest at ∼65% RH), a larger net water gain is realized (Frank, 1988). While it is important to note that this high humidity scenario is not tested in the current study, RER values decrease during the scotophase (Fig. 1E) when RH is higher (25%), suggesting that *P. eremicus* could be cueing into the higher humidity to preferentially oxidize lipids leading to a net metabolic gain in water.

### Sexual dimorphism

Interestingly, males and females responded differently to lack of water, with body temperature being the most notable difference. Females decreased their body temperature while males maintained their body temperature when compared with their hydrated counterparts. Whether this sexually dimorphic response is a strategy or a consequence is an open-ended question that cannot be answered using the data presented here; this response may be the product of high costs of reproduction in females, but not males. Indeed, similar patterns of sexual dimorphism in response to resource availability has been observed in other rodent species (Cranford, 1977; Randolph et al., 1977; Murray and Smith, 2012). Previous studies hypothesized that sexual dimorphism differences can be explained by differences in body size, metabolism, respiratory rate or activity (Cryan and Wolf, 2003). While we do not have direct measurements of respiratory rate or activity, the production of CO_2_ follows the patterns of EE, providing indirect yet strong evidence that respiratory and metabolic rates (EE) as well as activity are all sexually dimorphic, consistent with observations in humans (Glucksmann, 1974; Mittendorfer, 2005; Pomatto et al., 2018) and has also been observed in *P. eremicus* by McNab and Morrison (1963) and Colella et al. (2021b).

Male reproduction is mainly limited by access to females (Bateman, 1948), therefore, torpor or estivation by males could reduce male reproductive success. Furthermore, sperm quantity and quality is dependent on body temperature (Moore, 1926; Pérez-Crespo et al., 2008) and while typically resolved by externalizing the testes to the scrotum during excessive heat, a decrease in body temperature, as is seen in females (discussed below), could reduce sperm viability. Maintaining consistent body temperatures also allows for regular biological reactions, such as enzymatic processes and protein folding which have evolved to function best at a single temperature and can influence a series of functions not directly related to reproduction, such as growth rate, metabolic biorhythms and environmental sensing (Glucksmann, 1974; Hochachka and Somero, 2002; McPherson and Chenoweth, 2012; Calisi et al., 2018). Our data support this as body temperature was unchanged for dehydrated males compared with their hydrated counterparts for the entire experiment, suggesting a maintenance of reproductive investment at the cost of long-term survival.

In contrast, female reproduction is primarily limited by their access to resources (Bateman, 1948), in this case water. During the course of our study, female body temperature, EE and WLR all decreased, suggesting torpor and or estivation, consistent with MacMillen (1983). Specifically, homeostatic responses such as adaptive heterothermy, a process which reduces evaporation by storing body heat (1) reduces the air to body temperature gradient thus decreasing inward heat flow, (2) minimizes water loss from evaporative cooling (Schmidt-Nielsen et al., 1956; Schoen, 1972; Taylor, 1972; Cain et al., 2008). In small endotherms with high surface area to volume ratios, heterothermy can lead to substantial energy and water savings (Walsberg, 2000; Speakman and Król, 2010; Turbill and Stojanovski, 2018). For females, reproductive demands are especially high, particularly during pregnancy and in lactating females (not measured in this study; Sorensen et al., 2005; Murray and Smith, 2012), and minimizing energy costs or allocating pulses of resources to reproductive energy could increase reproductive success (Smith et al., 2014; Flores-Manzanero et al., 2019). Although homeostatic responses are quite common among endotherms (Boyles et al., 2011; 2013; Canale et al., 2012; McGuire et al., 2014; Dammhahn et al., 2017) and are essential for short term survival, they incur energetic, resource and/or fitness costs when the disturbance lasts longer than the homeostatic tolerance (Wingfield et al., 1992; Boonstra, 2004; Canale et al., 2012; McGuire et al., 2014; Dammhahn et al., 2017).

Although not statically significant, males with access to water weighed more than females with access to water at all time points, with males having higher body mass. After 72 h, water-deprived males did not weigh significantly more than females with access to water (Fig. 3). Differences in body composition, size or mass between males and females could potentially result in variation in how heat is conserved (McDonald et al., 1989). Animals with smaller body sizes (females in this experiment) exhibit an increased ratio of surface area to volume, making them more susceptible to both dehydration and hyperthermia than larger animals (males in this experiment) (Jessen, 2012; Smith and Lyons, 2011). Males may experience metabolic depression (Fig. 1D); however, their greater body mass may function as a buffer, potentially limiting the degree to which dehydration and hyperthermia are experienced compared with females.

### Electrolytes

In order to gain a deeper understanding of how water deprivation affects the physiological functioning of endotherms in desert environments, we collected serum electrolyte data from both males and females with and without access to water at the end of the experimental period. Electrolytes are essential for all physiological functions, including regulating fluid balance, transmitting nerve impulses and maintaining the acid–base balance (Hasona and Elasbali, 2016). Additionally, electrolyte levels can provide insight into an individual's overall metabolic state and renal function, and can be indicative of dehydration, kidney disease or heart failure (Kutscher, 1968; Cheuvront et al., 2010).

The kidney typically ensures that fluid and electrolyte balance remain within a narrow range, and this is conducive to efficient biochemical and physiological processes. Altered electrolytes, such as K, iCa and Na, are associated with dehydration (Abubakar and Sule, 2010; Cheuvront et al., 2010; Kutscher, 1968) and may result in fatigue, cognitive dysfunction and changes in osmotic pressure, which may affect blood pressure. More severe electrolyte abnormalities may cause cardiac arrhythmias and lead to death (Abubakar and Sule, 2010). We uncovered significant differences in electrolyte values between water treatments (Na, BUN, Hct, osmolality, Hb, Cl and total CO_2_) that were similar to those reported in Kordonowy et al. (2017), suggesting dehydration.

Despite being statistically insignificant, glucose trended downwards for males and females without water when compared with their hydrated counterparts. During fasting, blood glucose levels decrease because of a lack of glucose absorbed from the GI tract (Jensen et al., 2013). Previous studies have shown that the duration of fasting significantly affects blood glucose levels up to 72 h, but after 72 h there is no further decrease (Jensen et al., 2013). In humans, glucose concentrations are maintained regardless of the duration of starvation (Watford, 2015). Initially, carbohydrates are depleted during the first 24 h; however, during prolonged starvation gluconeogenesis provides glucose by breaking down skeletal muscle proteins (Watford, 2015). Although the current study did not measure food intake, glucose levels are being maintained, suggesting that the mice could be reducing food intake, consistent with other studies of dehydration anorexia in rodents and/or a shift toward increased glycogenolysis and lipolysis to maintain glucose concentrations (Salter and Watts, 2003; Schoorlemmer and Evered, 2002; Watts and Boyle, 2010), meaning that the liver is possibly serving as a buffer for blood glucose concentration.

### Conclusion

The extreme aridity of desert environments plays a role in shaping biological processes; however, the physiological mechanisms that allow animals to maintain salt and water homeostasis are still not well understood. Rapid climate change can challenge this tolerance. *Peromyscus eremicus* is native to the arid deserts in southwest North America. Past studies have shown that cactus mice are highly adapted to desert conditions, with efficient water retention and dehydration tolerance. Therefore, cactus mice represent an interesting experimental model to examine physiological adaptations and thresholds.

In this study, we explored the physiological mechanisms that enable cactus mice to survive in desert habitats. By integrating laboratory-based experiments with long-term physiological data collected from a captive colony of cactus mice in a simulated desert environment, we investigated their response to water deprivation. Our findings revealed that mice without access to water exhibit significantly lower energy expenditures, leading to reduced water loss compared with mice with access to water. Moreover, significant mass loss was observed during the first 24 h, likely attributed to dehydration anorexia, which is an adaptive response aimed at limiting fluid loss by reducing waste and the solute load while facilitating water reabsorption from the kidneys and gastrointestinal tract. Furthermore, our observations indicate a relationship between body temperature and sex. Males without access to water maintained their body temperature compared with hydrated males, whereas females without access to water experienced decreased body temperature, suggesting that the occurrence of daily torpor in females is an adaptive response that is likely to be related to reproductive investment.

By examining the physiological responses of water-deprived *P. eremicus*, we gain valuable insights into how adaptations developed over long evolutionary timescales. Given the current global climate change and the escalating desertification trends, it becomes imperative to investigate the plasticity and mechanisms of response in desert-adapted species. Such investigations hold the potential to enhance our understanding of organismal responses to the increasingly unpredictable climatic conditions.

## Supplementary Material

10.1242/jexbio.246386_sup1 Supplementary information

## ECR Spotlight

This article has an associated ECR Spotlight interview with Danielle Blumstein.
