# Supplementary material for: When the tap runs dry: the physiological effects of acute experimental dehydration in Peromyscus eremicus
Source: J Exp Biol. 2023 Dec 1;226(23):jeb246386. doi: 10.1242/jeb.246386 (PMC10714145; doi:10.1242/jeb.246386)
Supplement: Supplementary information [file jexbio-226-246386-s1.pdf]

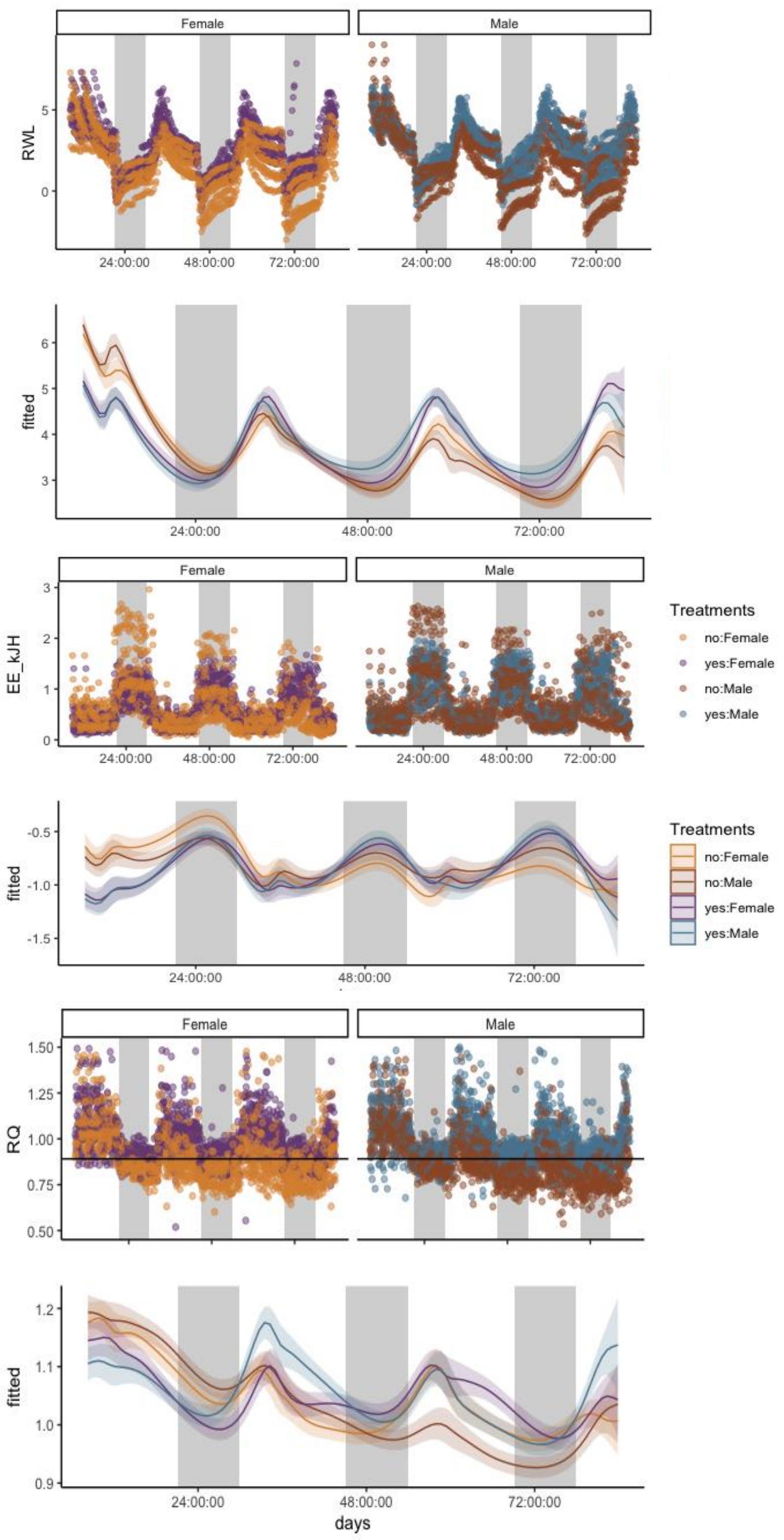

Journal of Experimental Biology • Supplementary information

Fig. S1. Raw plotted data (top) and general additive mixed models (GAMM) graph (lower) for water loss rate (WLR g h^{-1}), energy expenditure (EE kJ h^{-1}), and respiratory exchange rate (RER) for female and male *Peromyscus eremicus* with and without access to water. The smoothing curves for each response variable included two fixed effects; water treatment (yes vs no) and sex, two random effects; mouse identification number and date of data collection, and two regression terms: time in days and diurnal cycle. For the lower graph, the y-axis is the effect of the x-axis on WLR as estimated by a multivariable GAMM. Shaded areas are 95% confidence intervals.

Table S1. Generalized additive mixed models (GAMM) statical model and results for rate of water loss (WLR, H2O g/hr-1).

```
Formula:
H2Omg ~ H2O * Sex + s(days, time_in_D, by = tt, k = 70), data = dd, random =
list(startexp = ~1, Animal_ID = ~1|startexp), method="REML")

Parametric coefficients:
              Estimate Std. Error t value Pr(>|t|)
(Intercept)   1.67969      0.02029  82.775   <2e-16 ***
H2Oyes        0.83920      0.02874  29.195   <2e-16 ***
SexMale       0.27509      0.02878   9.557   <2e-16 ***
H2Oyes:SexMale  0.06247    0.04054   1.541     0.123
---
Signif. codes:  0 '***' 0.001 '**' 0.01 '*' 0.05 '.' 0.1 ' ' 1

Approximate significance of smooth terms:
                                  edf Ref.df    F p-value
s(days,time_in_D):ttno:Female   56.81  71.02 67.57  <2e-16 ***
s(days,time_in_D):ttyes:Female 63.72  78.17 55.76  <2e-16 ***
s(days,time_in_D):ttno:Male      62.10  76.51 66.08  <2e-16 ***
s(days,time_in_D):ttyes:Male   62.61  77.02 58.38  <2e-16 ***
---
Signif. codes:  0 '***' 0.001 '**' 0.01 '*' 0.05 '.' 0.1 ' ' 1

R-sq.(adj) =  0.764   Deviance explained = 77.3%
-REML = 8209.6  Scale est. = 0.65701   n = 6463
```

Table S2. Generalized additive mixed models (GAMM) statical model and results for measurements of energy expenditure (EE kJ/hr-1).

```
Formula:
log(EE_kJH) ~ H2O * Sex + s(days, time_in_D, by = tt, k = 70), data = dd, random =
list(startexp = ~1, Animal_ID = ~1|startexp), method="REML")
 Parametric coefficients:
              Estimate Std. Error t value Pr(>|t|)
(Intercept)   -0.75378     0.01067 -70.658  < 2e-16 ***
H2Oyes        0.07460      0.01511   4.937 8.12e-07 ***
SexMale       0.21041      0.01511  13.925  < 2e-16 ***
H2Oyes:SexMale -0.04238    0.02128  -1.992   0.0464 *
---
Signif. codes:  0 '***' 0.001 '**' 0.01 '*' 0.05 '.' 0.1 ' ' 1

Approximate significance of smooth terms:
                                  edf Ref.df    F p-value
s(days,time_in_D):ttno:Female   43.79  53.83 42.03  <2e-16 ***
s(days,time_in_D):ttyes:Female 42.67  52.67 45.47  <2e-16 ***
s(days,time_in_D):ttno:Male      42.46  52.42 33.68  <2e-16 ***
s(days,time_in_D):ttyes:Male   46.23  56.15 53.83  <2e-16 ***
---
Signif. codes:  0 '***' 0.001 '**' 0.01 '*' 0.05 '.' 0.1 ' ' 1

R-sq.(adj) =  0.603   Deviance explained = 61.4%
-REML = 3951.7  Scale est. = 0.18183   n = 6463
```

Journal of Experimental Biology • Supplementary information

Table S3. Generalized additive mixed models (GAMM) statical model and results for respiratory exchange ratio (RER).

```
Formula:
RQ ~ H2O * Sex + s(days, time_in_D, by = tt, k = 70), data = dd, random =
list(startexp = ~1, Animal_ID = ~1|startexp), method="REML")

Parametric coefficients:
             Estimate Std. Error t value Pr(>|t|)
(Intercept)  0.911886   0.002997 304.246  < 2e-16 ***
H2Oyes       0.079386   0.004245  18.702  < 2e-16 ***
SexMale     -0.023301   0.004243  -5.492 4.14e-08 ***
H2Oyes:SexMale  0.031496   0.005976   5.271 1.41e-07 ***
---
Signif. codes:  0 '***' 0.001 '**' 0.01 '*' 0.05 '.' 0.1 ' ' 1

Approximate significance of smooth terms:
                                  edf Ref.df     F p-value
s(days,time_in_D):ttno:Female   37.40  47.03 20.25  <2e-16 ***
s(days,time_in_D):ttyes:Female  37.59  47.22 17.27  <2e-16 ***
s(days,time_in_D):ttno:Male     29.41  37.68 27.09  <2e-16 ***
s(days,time_in_D):ttyes:Male    37.85  47.46 16.95  <2e-16 ***
---
Signif. codes:  0 '***' 0.001 '**' 0.01 '*' 0.05 '.' 0.1 ' ' 1

R-sq.(adj) =   0.42   Deviance explained = 43.3%
-REML = -4299.2  Scale est. = 0.014362  n = 6463
```

Journal of Experimental Biology • Supplementary information
